# Amplifying the signal of localized surface plasmon resonance sensing for the sensitive detection of Escherichia coli O157:H7

**DOI:** 10.1038/s41598-017-03495-1

**Published:** 2017-06-12

**Authors:** Liping Song, Lei Zhang, Youju Huang, Liming Chen, Ganggang Zhang, Zheyu Shen, Jiawei Zhang, Zhidong Xiao, Tao Chen

**Affiliations:** 1 Department of Chemistry, Huazhong Agricultural University, Wuhan, 430070 China; 2 Key Laboratory of Marine Materials and Related Technologies, Zhejiang Key Laboratory of Marine Materials and Protective Technologies, Ningbo Institute of Materials Technology and Engineering, No. 1219 Zhongguan West Road, Ningbo, 315201 China

## Abstract

Gold nanorods (Au NRs) based localized surface plasmon resonance (LSPR) sensors have been widely employed in various fields including biology, environment and food safety detection, but their size- and shape-dependent sensitivity limits their practical applications in sensing and biological detection. In our present work, we proposed an approach to maximally amplify the signal of Au NRs based LSPR sensing by coating an optimized thickness of mesoporous silica onto Au NRs. The plasmonic peaks of Au NRs@SiO_2_ with different shell thickness showed finely linear response to the change of surrounding refractive index. The optimized thickness of mesoporous silica of Au NRs@SiO_2_ not only provided high stability for LSPR sensor,but also displayed much higher sensitivity (390 nm/RIU) than values of Au NRs from previous reports. The obtained Au NRs@SiO_2_ based LSPR sensor was further used in practical application for selectively detection of the *E. coli* O157:H7, and the detection limit achieved 10 CFU, which is much lower than conventional methods such as electrochemical methods and lateral-flow immunochromatography.

## Introduction

The localized surface plasmon resonance (LSPR) is a spectroscopic phenomenon based on the resonant oscillations of free electrons of various materials including noble metal nanoparticles, Al nanoparticles, conventional semiconductors and 2D materials, when stimulated by incident light^1–3^. Except the size, shape^4, 5^ and composition of nanoparticles, the frequency and intensity of the LSPR bands are sensitive to the interparticle spacingno and dielectric environment^6, 7^. Therefore, the response of plasmonic nanoparticles to the refractive index variation of surrounding medium is employed to develop LSPR sensors in broad fields of biology, food, and environment^8–11^. However, due to the low detection sensitivity and capacity^7, 12, 13^ and poor stability, the development of LSPR sensors is limited, especially in practical applications. For example, numerous efforts have been devoted to develop various methods for the detection of *E. coli* O157:H7, which is a gram-negative enteric bacteria, causing severe intestinal infections in humans^14, 15^. Traditional methods ^16–18^ such as electrochemical methods and lateral-flow immunochromatography for detecting *E. coli* O157:H7 are always time-consuming and the sensitivity is not very high^19–21^ and limits their practical use as a commercial product. The objective of the present study is to develop a high performance LSPR sensor for practical application in real sample analysis (*E. coli* O157:H7) that would achieve high sensitivity and stability.

In recent years, some strategies were suggested to improve the sensitivity and stability of LSPR sensors such as morphology optimization of nanoparticles, surface functionalization and single nanoparticle detection^22, 23^. Meanwhile, surface functionalization served as a powerful means not only to effectively improve sensitivity and stability of LSPR sensors but also flexibly modify plasmonic nanoparticles with desired functions for further applications^24^. For example, polymer or biomolecules coated Au nanoparticles LSPR sensors show higher selectivity and stability^25^.

Homogeneous mesoporous silica has been proven to be a versatile biomaterial to directly improve stability of nanoparticles in complex bio-system, due to its excellent biocompatibility. On the other hand, mesoporous structure^26–28^ provides large-area and easy-decorated surface for maximally capturing the target molecules to improve the sensitivity of LSPR. Since the encapsulated SiO_2_ determines the refractive index of surrounding environment, the thickness of SiO_2_ attributed to a vital parameter to affect LSPR sensitivity. However, there is a controversial point about the effect of thickness of SiO_2_ shell on LSPR sensitivity. For instance, Xu and co-workers synthesize 8 nm mesoporous silica shell coated Au NRs to improve the sensitivity to 325 nm/RIU^22^. Wang and co-workers used 21 nm silica coated Au NRs to amplify the sensitivity for detecting biomolecules^29^. Moreover, Huang and co-workers considered that a 2–3 nm silica shell coated on Au NRs induces a highest sensitivity for plasmonic organic photovoltaic devices^30^. The recent theoretical calculation reveals that ultrathin silica coating (within 3.5 nm) would be ideal for improving the sensitivity^31^. Therefore, the aim of our present work is to explore the cut-off thickness in a small size, and further amplify the sensitivity of Au NRs maximally for practical application for detection of *E. coli* O157:H7.

Herein, a series of Au nanorods (Au NRs) with different mesoporous silica shell are synthesized to study the effects of silica shell thickness on the sensitivity of Au NRs LSPR symmetrically. The as-prepared Au NRs@SiO_2_ with optimal shell thickness showed high sensitivity and used as efficient LSPR biosensor to detect *E. coli* O157:H7. Au NRs with elongated geometry were selected as plamonic nanoparticles for LSPR sensor due to their tunable and broad spectra range from the visible to near-infrared region^24, 32, 33^. The thickness of SiO_2_ on AuNRs was optimized by adjusting the concentration of Cetyltrimethyl Ammonium Bromide (CTAB) and the amount of tetraethyl orthosilicate (TEOS), achieving sensitive Au NRs@SiO_2_. To verify the performance of Au NRs@SiO_2_ as LSPR sensors, Au NRs@SiO_2_ with optimal thickness was modified with specific antibody to selectively detect *E. coli* O157:H7 in high sensitivity. The detection limit of Au NRs@SiO_2_ for *E. coli* O157:H7 is lower than 10 Colony-Forming Units (CFU), which is superior over the values of traditional methods^34, 35^.

## Results and Discussion

**Figure 1:**
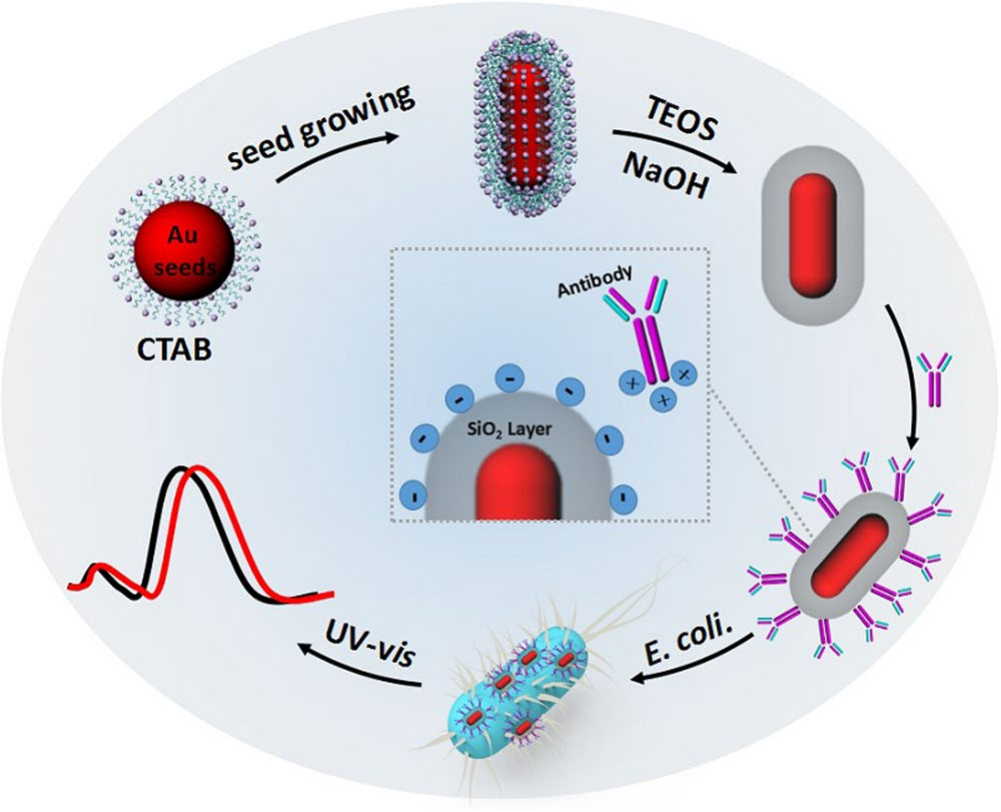
Schematic illustration of the synthesis of Au NRs and Au NRs@SiO_2_ and the procedure of detection for *E. coli* O157:H7.

**Figure 2:**
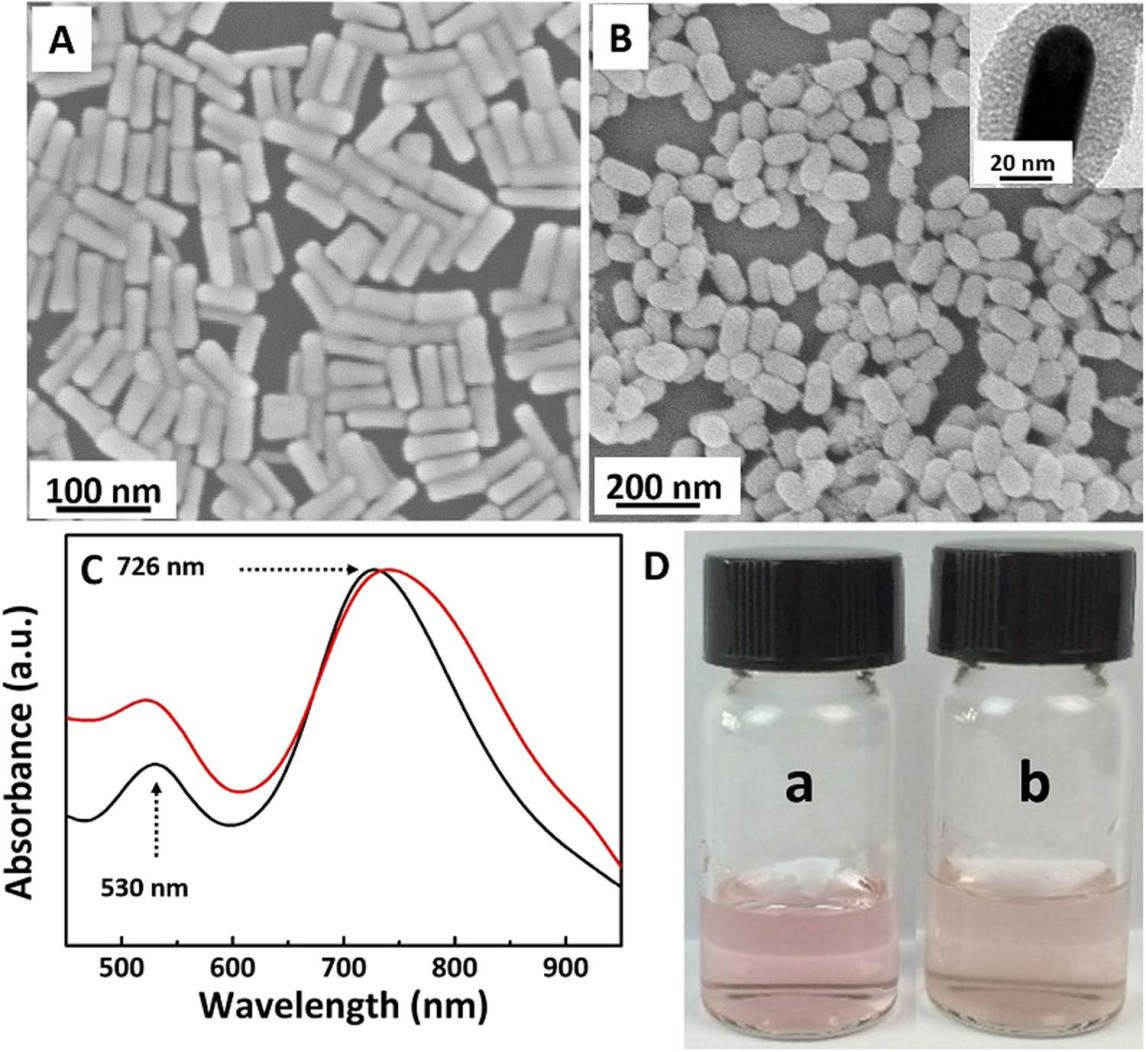
SEM (**A**) and TEM (**B**) of as-prepared Au NRs and Au NRs@SiO_2_, respectively; (**C**) UV-vis spectra of Au NRs (black) and Au@SiO_2_ (red); (**D**) Photographs of Au NRs (a) and Au@SiO_2_ (b).

Au NRs (Fig. 1 and 2A) were synthesized by using the seed-mediated growth method^36^, which has an aspect ratio of 2.9. As prepared Au NRs showed typical horizontal and longitudinal LSPR bands at 530 nm and 726 nm, respectively (Fig. 2C). The number of Au NRs in solution was estimated to be 2.1 nM according to its extinction coefficient (3.9 ± 0.5 × 10^9^ M^−1^ cm^−1^) at the longitudinal plasmon peak (726 nm)^37, 38^. According to the traditional method (soft-templating method), Au NRs were uniformly coated with mesoporous silica, forming monodispersed core-shell AuNRs@SiO_2_ nanostructures (Fig. 1 and 2B)^39^. In the process of silica coating, CTAB formed a bilayer around Au NRs, which served as organic template for the formation of mesoporous silica shell^40^. Moreover, it was found that the longitudinal LSPR band of Au NRs@SiO_2_ redshifted clearly compared with Au NRs (Fig. 2C), leading to a lighter pink color (illustration of Fig. 2D).

**Figure 3:**
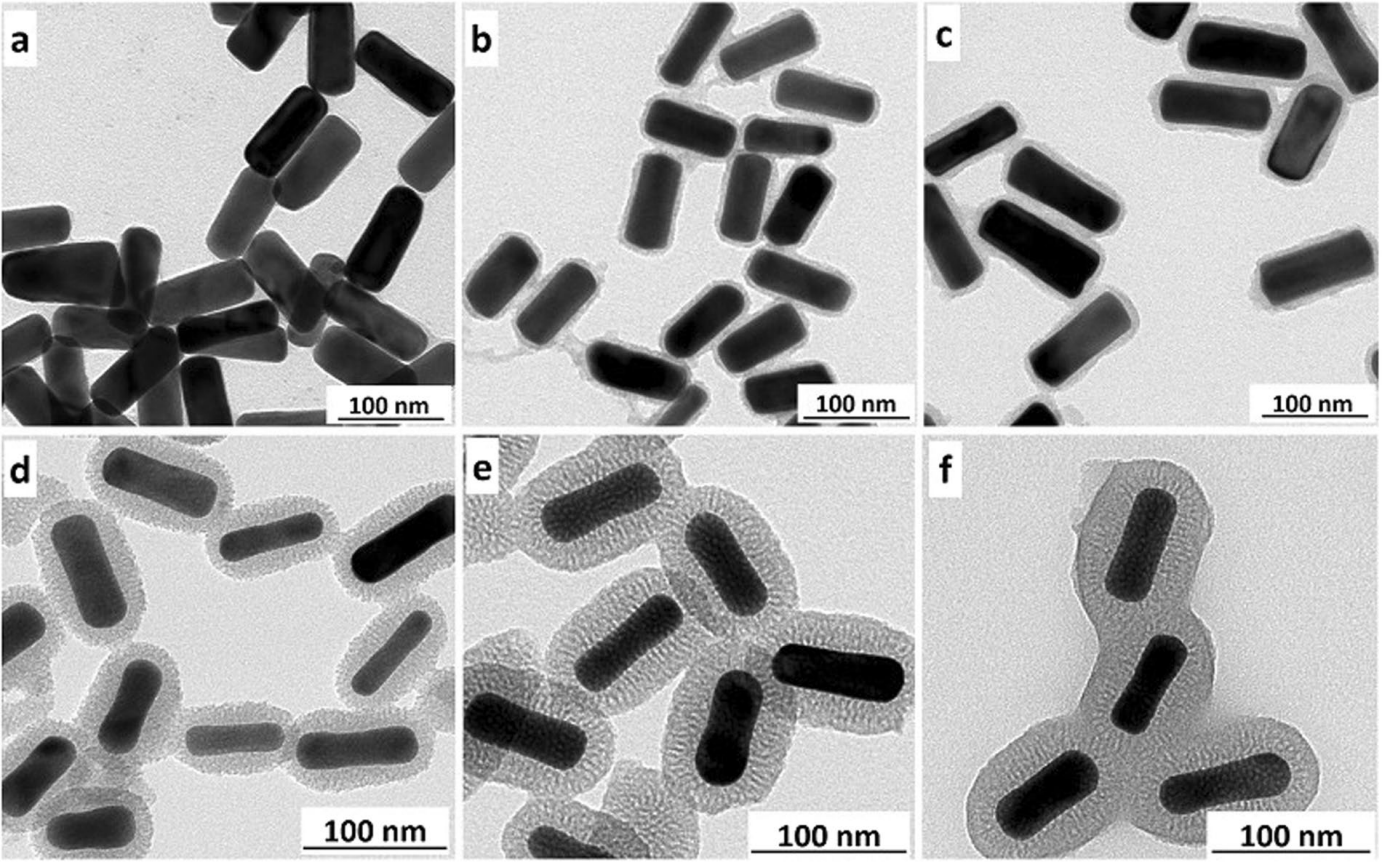
TEM images of Au NRs@SiO_2_ with different shell thickness. The thickness of silica were as follows, respectively: (**a**) 2 nm; (**b**) 5 nm; (**c**) 10 nm; (**d**) 15 nm; (**e**) 20 nm; (**f**) 25 nm.

In order to study systematically the influence of silica thickness on the LSPR of Au NRs@SiO_2_, Au NRs@SiO_2_ with different shell thickness were prepared by adjusting the concentration of CTAB and the amount of TEOS. When the additive amount of TEOS was changed from 2 µL to 20 µL, the silica shell thickness increased from 2 nm to 25 nm (Fig. 3). It is noteworthy that the thickness of silica was calculated according to the general theory of statistics by measuring more than 100 particles. Fig. S1 showed the statistical values and statistical distribution of Au NRs@SiO_2_ with 2 nm SiO_2_ layer. The thickness of other SiO_2_ layers on Au NRs@SiO_2_ (5, 10, 15, 20, 25 nm) were also confirmed by the general theory of statistics. CTAB was used as the template for depositing silica and many previous reports showed that lower concentration of CTAB resulting in thicker silica shell^41–43^. Therefore, by adjusting the concentration of CTAB and the amount of TEOS, Au NRs@SiO_2_ with different shell thickness could be prepared. The uniform silica shell endows a protective layer on the surface of Au NRs, leading to extremely stable Au NRs dispersion that could keep for three months at room temperature even after removal of CTAB.

**Figure 4:**
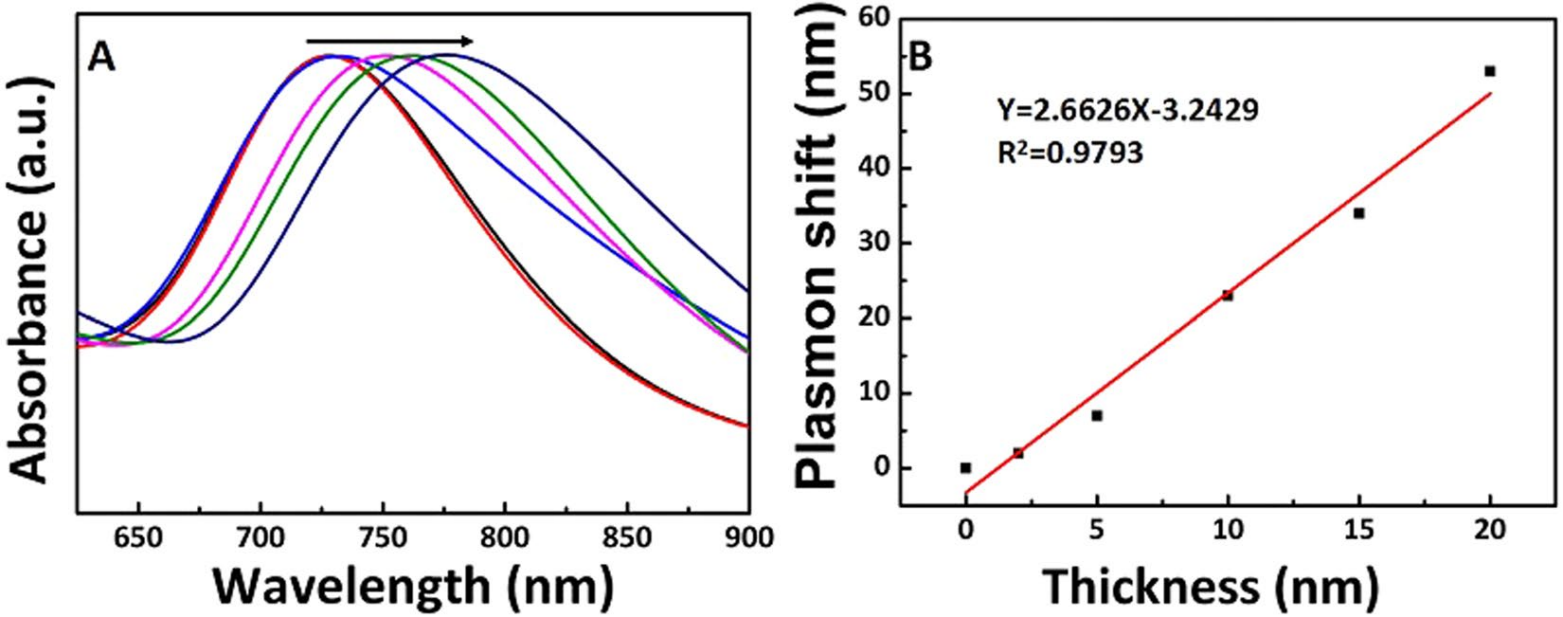
(**A**) Normalized extinction spectra of Au NRs@SiO_2_ with different shell thickness from 2 nm to 20 nm; (**B**) Linearity curve of plasmon shift vs. the thickness of silica.

Furthermore, the LSPR properties of Au NRs with different mesoporous silica shell were investigated by UV-*vis* absorption spectra (Fig. 4). The longitudinal plasmon bands of Au NRs@SiO_2_ redshifted gradually with increasing the thickness of silica shell (Fig. 4A). The relationship between plasmon shift and thickness of silica is shown in Fig. 4B, indicating that the plasmon band shift showed approximately linearly with increasing shell thickness. This was ascribed to the refractive index variation of silica shell when changing the thickness^27^. The refractive index of porous silica is much higher than the value of water. When the porous silica was coated onto the surfaces of Au NRs, the refractive index of surrounding environment of Au NRs increased, which was in accordance with previous work^44^. Therefore, the coating of silica on Au NRs led to redshift of the longitudinal plasmon band, which proved that the Au NRs@SiO_2_ was much sensitive to the change of surrounding refractive index than Au NRs. This allowed Au NRs@SiO_2_ could be employed as a LSPR sensor based on the redshift of longitudinal plasmon band induced by coating with mesoporous silica.

**Figure 5:**
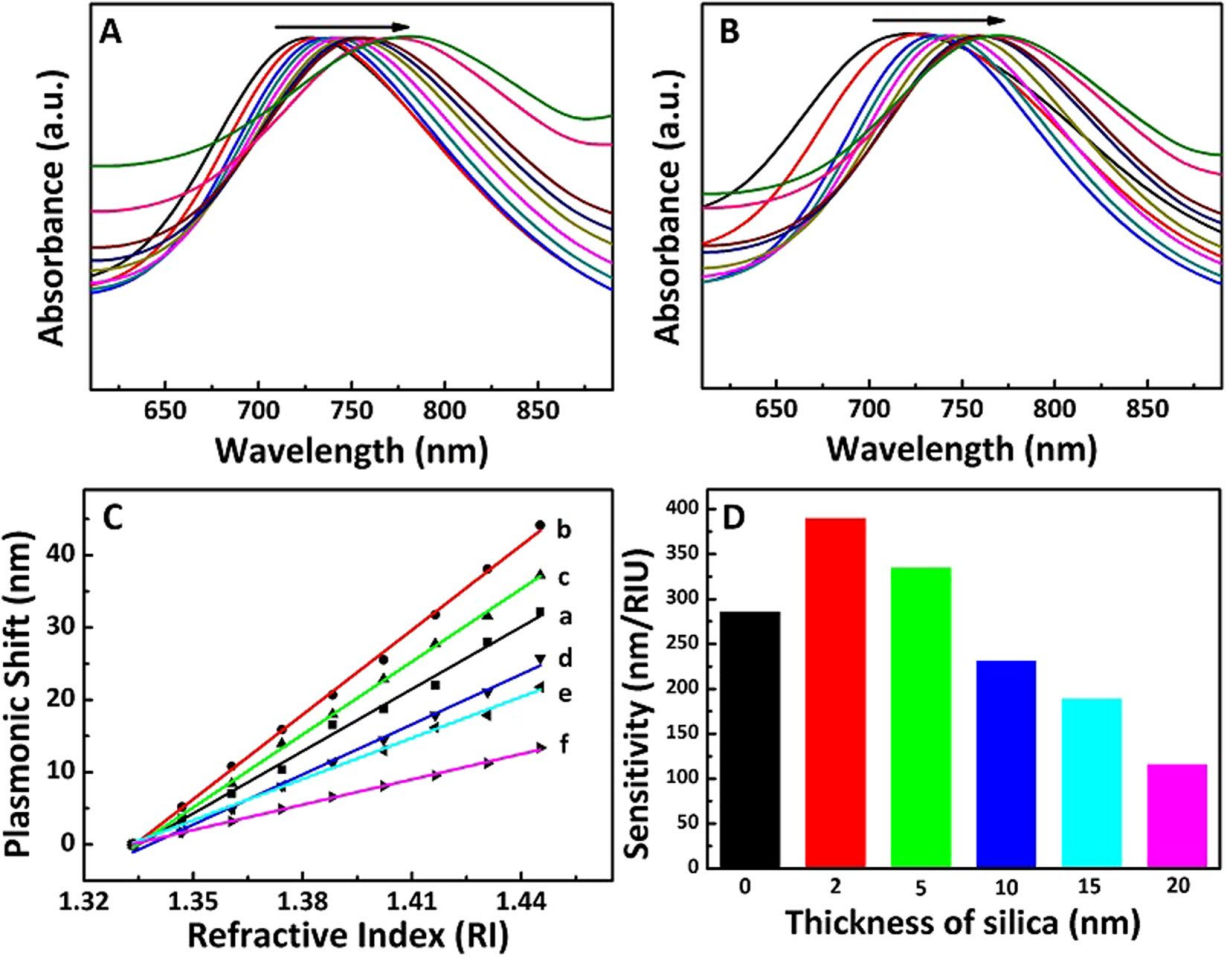
Normalized extinction spectra of Au NRs (**A**) and Au NRs@SiO_2_ with 2 nm shell thickness (**B**) in a mixture of water and glycerol with different volume ratios from 0.9 to 0.1 with the volume ratio of 0.1 interval; (**C**) Comparison of the refractive index sensitivity of Au NRs (a) and Au NRs@SiO_2_ with different SiO_2_ thickness from 2 nm to 20 nm (cross ponding to b to f); (**D**) Difference in sensitivity of Au NRs and Au NRs@SiO_2_ with different SiO_2_ thickness from 2 nm to 20 nm.

To verify the performance of the Au NRs@SiO_2_ based LSPR biosensor, Au NRs@SiO_2_ was firstly used to test the surrounding refractive index variations, as shown in Fig. 5. The sensitivity was calculation according to the universal experienced formula^45^:

1 $$\bigtriangleup \lambda ={\rm{m}}\cdot \bigtriangleup {\rm{n}}$$

Δλ is the difference of the LSPR wavelength before and after coating; m is the sensitivity, Δn is the change in the refractive index.

The refractive index was tuned by adjusting the volume ratio of water and glycerol (from 0.9 to 0.1 with the volume ratio of 0.1 interval), and the refractive indices of the mixture solvents were calculated based on the Lorentz-Lorenz equation, as shown in the experimental details^23, 46^. Both of Au NRs and Au NRs@SiO_2_ exhibited band redshift when increasing the volume ratio of glycerol to water (Fig. 5A and B).

The plasmon peaks of Au NRs and Au NRs@SiO_2_ in mixed solution was plotted against with refractive index, as shown in Fig. 5C. It was found that plasmon absorption shifts linearly to longer wavelength with increasing the refractive index^22, 47, 48^. In addition, only absorption is considered here and the influence of scattering are neglected due to higher absorption than scattering of nanorods^49^.

The slope indicated the refractive index sensitivities, as shown in Fig. 5D. The sensitivity values of Au NRs@SiO_2_ with different thickness were ranged from 110 nm/RIU to 390 nm/RIU. The Au NRs@SiO_2_ with 2 nm shell exhibited highest sensitivity approaching to 390 nm/RIU, which was much higher than that of previous reports (the reported highest value was 325 nm/RIU)^22, 33, 50^. The sensitivity of Au NRs@SiO_2_ had intimate relation with the silica shell and reduced gradually with increasing the shell thickness, which agreed with the results in previous work^22, 31^. The sensitivity of Au NRs@SiO_2_ with 2 nm or 5 nm shell was larger than the values of Au NRs and the Au NRs@SiO_2_ with other thickness. Generally, the improvement in sensitivity can be explained according to the universal experienced formula^50–53^

2 $$\bigtriangleup {\rm{\lambda }}={\rm{m}}({n}_{adsorbate}-{n}_{medium})(1-{e}^{-\frac{2d}{{l}_{d}}})\,$$

Δλ is the difference of the LSPR wavelength before and after coating; m is the sensitivity; n_adsorbate_ and n_medium_ are the refractive indexes of the shell and the solution, respectively; d is the thickness of the coating shell and l_d_ is the electromagnetic field decay length of the system, make A equal to $(1-{e}^{-\frac{2d}{{l}_{d}}})$.

When Au NRs coated with thin silica, the change of A was ignored due to the minor change of d. The sensitivity was determined by the value of Δλ/Δn, which resulted in higher sensitivity than Au NRs due to higher change of Δλ than Δn. However, when the silica shell became much thicker, the change of *d* can’t be ignored, which resulted in the increase of A. Though the value of Δλ increased, the sensitivity decreased following the increase of Δn and A. Therefore, it is not uncommon that the Au NRs@SiO_2_ with 2 nm shell was with the highest sensitivity for about 390 nm/RIU.

**Figure 6:**
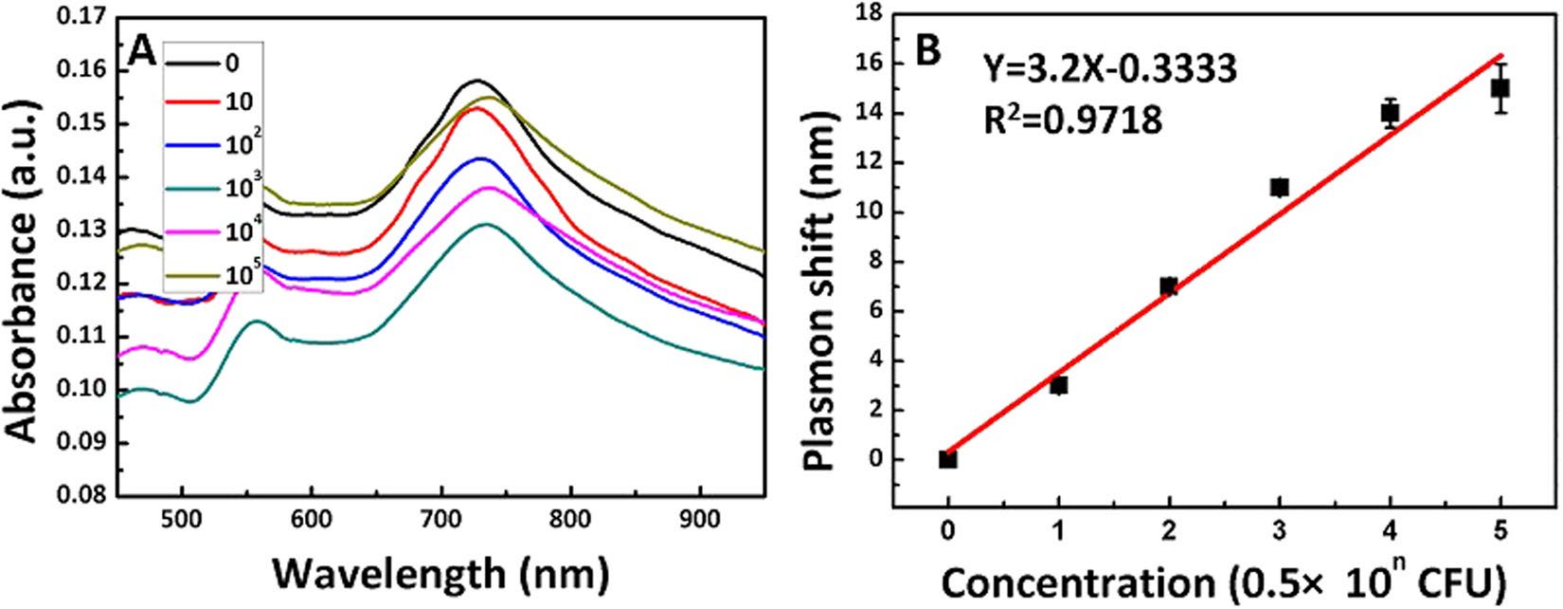
(**A**) UV-vis spectra of Au NRs@SiO_2_ after reaction with different concentrations (from 0 to 0.5 × 10^6^ CFU) of *E. coli* O157:H7 in the 0.01 M PBS solutions (pH = 7.4); (**B**) Linearity curve for the plot of plasmon shift vs. the logarithm of concentration of *E. coli* O157:H7 (n = 1, 2, 3, 4, 5, 6).

Au NRs@SiO_2_ with 2 nm of shell thickness showed optimal sensitivity of 390 nm/RIU and can serve as ideal candidate for high-efficiency LSPR sensors. Au NRs@SiO_2_ was modified with specific antibody to selectively detect *E. coli* O157:H7. Due to the negative shell, the surface of the Au NRs@SiO_2_ was negatively charged, which provided opportunity for the electrostatic interaction between Au NRs@SiO_2_ and the positive antibody (Fig. S2) and simplified the procedure and avoided extra chemical treatment. *E. coli* O157:H7 samples with different concentration were mixed with antibody-conjugated Au NRs@SiO_2_ to promote the sufficient incubation. Then the typical plasmon band shifts of Au NRs were measured using UV-*vis* absorption spectra to detect *E. coli* O157:H7 (Fig. 6A). After integrated with *E. coli* O157:H7, the longitudinal plasmon band of Au NRs@SiO_2_ redshifted gradually and the absorption intensity was decreased gradually when increasing the concentration of *E. coli* O157:H7 in the range from 0 to 0.5 × 10^5^ CFU (Fig. 6A).

Au NRs@SiO_2_ was sensitive to the surrounding refractive. With increasing the concentration of *E. coli* O157:H7, the refractive index around Au NRs@SiO_2_ increased, which led to the redshift of the longitudinal plasmon band^24^. The relationship between plasmon band shift and logarithmic concentration of *E. coli* O157:H7 is shown in Fig. 6B. It was found that the redshift changed linearly with the logarithmic concentration of *E. coli* O157:H7, which made it possible to detect *E. coli* O157:H7 quantitatively. Furthermore, the experiment of the selectivity of the sensor had been done by using Salmonella Typhimurium (S. Typhimurium) with the antibody of *E. coli* O157:H7 (Fig. S3) to replace *E. coli* O157:H7. The LSPR band of Au NRs@SiO_2_ was almost unchanged except some negligible change (around 0.5 nm). The results indicated that the antibody-conjugated Au NRs@SiO_2_ only bind to *E. coli* O157:H7 due to the specific recognition.

## Conclusion

In summary, a highly sensitive plasmon resonance biosensor based on Au NRs@SiO_2_ was prepared to detect *E. coli* O157:H7 quickly and simply. By adjusting the concentration of CTAB and the amounts of TEOS, we synthesized monodisperse Au NRs@SiO_2_ with serious uniform silica shell. Meanwhile, the refractive index sensitivities of both Au NRs and Au NRs@SiO_2_ with different thickness of silica shell were investigated and the Au NRs@SiO_2_ with 2 nm shell was most sensitive compared to others. In addition, the Au NRs@SiO_2_ with 2 nm shell was used here to detect the *E. coli* O157:H7 by simply physical adsorption between antibodies and Au NRs@SiO_2_. The results indicated that the plasmon shift was very sensitive to the change of the concentration of *E. coli* O157:H7 and monitoring *E. coli* O157:H7 at concentration lower than 10 CFU in less than 40 min, which meant the LSPR platform based on Au NRs@SiO_2_ was sensitive, simple, quick for monitoring *E. coli* O157:H7 without any expensive instruments and this label-free method was promising to applied in detecting pathogenic agents.

## Methods

### Reagents and materials

Hexadecyltrimethylammonium bromide (CTAB), Sodium borohydride (NaBH_4_) and Tetraethylorthosilicate (TEOS) were commercially available from Sigma-Aldrich Chemical Co. (St. Louis, MO, USA). L-Ascorbic Acid (AA) was purchased from Energy Chemical in Shanghai. Chloroauricacid (HAuCl_4_•3H_2_O, 99.9%), Silver nitrate (AgNO_3_), ethanol, hydrochloric acid (HCl) and sodium hydroxide were purchased from Sinopharm Chemical Reagent Co. Ltd. (Shanghai). *E. coli* O157:H7 (1.7 × 10^8^ CFU/mL), Salmonella Typhimurium (S. Typhimurium) (2.48 × 10^8^ CFU/mL) and the murine anti- *E. coli* O157:H7 monoclonal antibody were purchased from Meridian Life Science, Inc. (Memphis, TN). Phosphate buffer saline (PBS, pH = 7.4) was purchased from Sigma Chemical Company (St. Louis, MO) and was used to dilute the *E. coli* O157:H7 stock solution (1.7 × 10^8^ CFU/mL) with different concentrations (from 10 to 10^5^ CFU/mL). Other chemicals were purchased from Sinopharm Chemical Reagent Co., Ltd. (China) and used without any further purification.

Transmission electron microscopy (TEM) was performed on a JEOL JEM-2100F instrument and operated at 200 kV. Scanning electronic microscopy (SEM) measurements were carried out by a JEOL JMS-6700F scanning microscope. UV-*vis* absorption spectra were collected by virtue of TU-1810 UV-*vis* spectrophotometer provided by Purkinje General. The surface zeta potential data was performed on a Zeta Potential Analyzer.

### The Synthesis of Gold Nanorods

CTAB stabilized gold nanorods were synthesized using the seed-mediated growth method with minor modifications^36, 54^. First, 0.6 mL of freshly prepared ice-cold aqueous NaBH_4_ solution (0.01 M), was added into mixed aqueous solution containing 0.25 mL, 0.01 M HAuCl_4_ and 9.75 mL, 0.1 M CTAB. After string violently for 2 min, the seeds formed and were used within 10~120 min. The growth solution was prepared by mixing the aqueous solution of 4 mL, 0.01 M HAuCl_4_, 0.8 mL, 0.01 M AgNO_3_, 80 mL and 0.1 M CTAB firstly. Then 0.64 mL, 0.1 M freshly prepared aqueous ascorbic acid solution was added into the above mixture solution with gentle string, followed by adding 1.6 mL, 0.1 M HCl aqueous solution. After mixing the resultant solution with gentle string, 0.02 mL seed solution was added then mixing it with gentle inversion for 10 s and then left the growth solution undisturbed at least 6 h.

### The synthesis of Au NRs@SiO_2_ with different thicknesses

The synthesis of Au NRs@SiO_2_ with homogeneous silica thickness was carried out according to the traditional soft-templating method^55^. The 10 mL of as-prepared Au NRs was washed using deionized water by centrifugation (6500 rpm, 10 min) for 2 times. The supernatant was removed and the pellet was diluted to 10 mL by adding deionized water. Then a certain amount of NaOH (0.1 M) was added to the above solution to adjust the solution PH to 10.6. After the solution was mixed for 20 min, three 2 µL, 4 µL, 6 µL, 10 µL, 15 µL, 20 µL injections of 20% TEOS in ethanol solution was added under gentle stirring at 30 min intervals and the solution was mixed under room temperature for 24 h.

### Refractive Index Sensitivity Measurements

Water/glycerol solutions with a percentage of glycerol ranging from 0 to 90% at 10% intervals were used to change the refractive index experienced by the Au NRs and Au NRs@SiO_2_. The refractive indexes were calculated according to the Lorentz-Lorenz equation as follows:

3 $$\frac{{n}_{12}^{2}-1}{{n}_{12}^{2}+2}={\phi }_{1}\frac{{n}_{1}^{2}-1}{{n}_{1}^{2}+2}+{\phi }_{2}\frac{{n}_{2}^{2}-1}{{n}_{2}^{2}+2}$$

Wherein n_12_ is the refractive index of the mixture, n_1_ (1.3334) and n_2_ (1.4746) are the refractive indices of water and glycerol, respectively, *φ*_1_ and *φ*_2_ are their volume fractions. For sensitivity measurements, 3 mL particle samples in solvent with different concentration of glycerol were used. The location of the absorption peak changed response to the concentration variety of glycerol. The LSPR shift showed linearly dependence on the refractive index change and the slope represented the sensitivity of the corresponding sample.

### Immobilization of antibodies on Au NRs@SiO_2_ and Detection of *E. coli* O157:H7

Firstly, 5 mL of as-prepared Au NRs@SiO_2_ was washed for 2 times by centrifugation (6500 rpm, 6 min) and redispersed into 5 mL PBS solution (pH 7.4, 0.01 M). Then 1 mL 0.1 µg mL^−1^ antibodies for *E. coli* O157:H7 was added into the above solution and stirred for 30 min at room temperature. Then the antibodies conjugated Au NRs@SiO_2_ was centrifuged for once to remove free antibodies and redispered into 5 mL of PBS (pH 7.4, 0.01 M). 0.5 mL *E. coli* O157:H7 with different concentrations were mixed with 0.5 mL the as-synthesized antibodies conjugated Au NRs@SiO_2_. After gentle shaking for 10 min, the mixture was settled for 70 min without disturbance at 37 °C. Then the resultant solution was detected under UV-*vis* measurement. The selective experiment was similar to the above detecting experiment just replaced the *E. coli* O157:H7 with S. Typhimurium of equal volume.

## Electronic supplementary material

SR-supporting information
