# Supplementary material for: Amplifying the signal of localized surface plasmon resonance sensing for the sensitive detection of Escherichia coli O157:H7
Source: Sci Rep. 2017 Jun 12;7:3288. doi: 10.1038/s41598-017-03495-1 (PMC5468277; doi:10.1038/s41598-017-03495-1)
Supplement: Supplementary file 1 — SR-supporting information [file 41598_2017_3495_MOESM1_ESM.doc]

**Supporting Information:**

**Amplifying the signal of localized surface plasmon resonance sensing for the sensitive detection of Escherichia coli O157:H7**

Liping Song,^1,2^ Lei Zhang,^2^ Youju Huang,^2^,* Liming Chen,^2^ Ganggang Zhang,^2^ Zheyu Shen,^2^ Jiawei Zhang,^2^ Zhidong Xiao^1^,* and Tao Chen^2^,*

^1^ Department of Chemistry, Huazhong Agricultural University, Wuhan, 430070, China. ^2^ Division of Polymer and Composite Materials, Ningbo Institute of Material Technology and Engineering Chinese Academy of Sciences, No. 1219 Zhongguan West Road, Zhenhai District, Ningbo 315201, China. Fax: (0086)0574-87603570

E-mail: [yjhuang@nimte.ac.cn](mailto:yjhuang@nimte.ac.cn); [zdxiao@hzau.edu.cn](mailto:zdxiao@hzau.edu.cn) and [tao.chen@nimte.ac.cn](mailto:tao.chen@nimte.ac.cn).


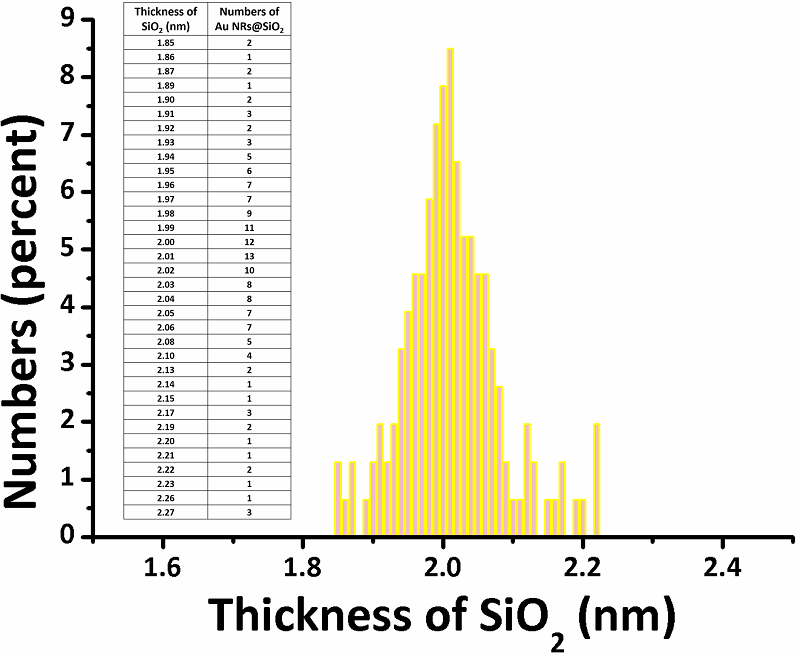


Figure S1 The statistical values and statistical distribution of Au NRs@SiO_2_ with 2 nm SiO_2_ layer.

**
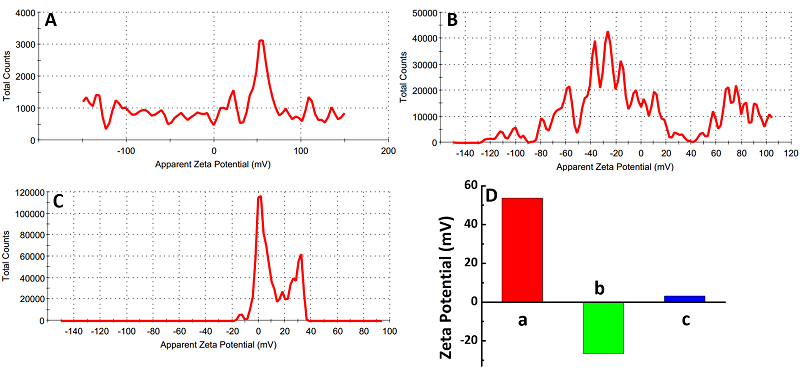
**

Figure S2. The surface zeta potential characterization of the Au NRs (A), Au NRs@SiO_2_ before (B) and after (C) antibody functionalization. (D) The histogram of the surface zeta potential characterization of the Au NRs (a), Au NRs@SiO_2_ before (b) and after (c) antibody functionalization.


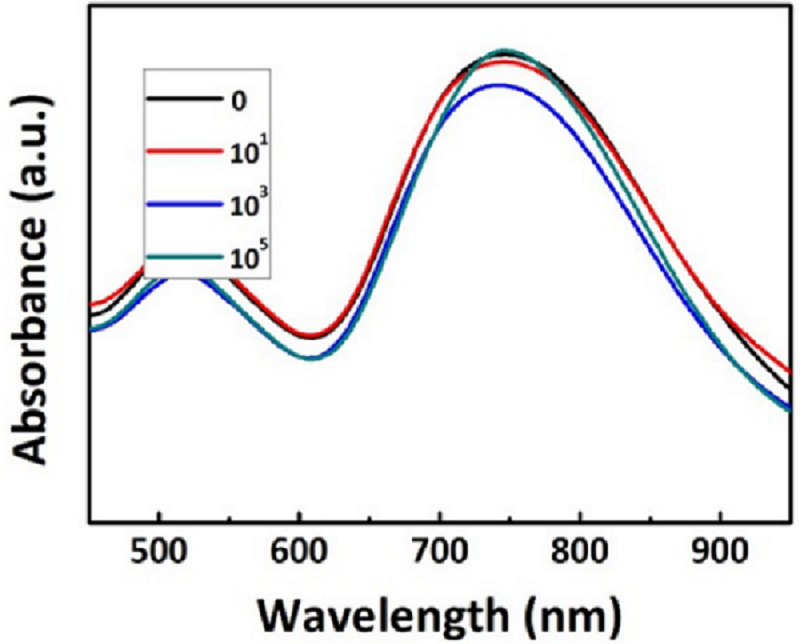


Figure S3. UV-*vis* spectra of antibody (anti-*E. Coli* O157:H7) conjugated Au NRs@SiO_2_ after reaction with different concentrations (from 0 to 0.5×10^5^ CFU) of S. Typhimurium in the 0.01 M PBS solutions (pH=7.4).
